# Poly[(acetato-κ^2^ *O*,*O*′)aqua­(μ_4_-1*H*-benzimidazole-5,6-dicarboxyl­ato-κ^5^ *N* ^3^:*O* ^5^,*O* ^5′^:*O* ^5^,*O* ^6^:*O* ^6′^)praseodymium(III)]

**DOI:** 10.1107/S1600536810036986

**Published:** 2010-09-25

**Authors:** Zi-Yu Pan, Jin-Hua Chen, Jian-Fen Lin, Xuan Xu, Yi-Fan Luo

**Affiliations:** a School of Chemistry and Environment, South China Normal University, Guangzhou 510006, People’s Republic of China

## Abstract

In the title complex, [Pr(C_9_H_4_N_2_O_4_)(C_2_H_3_O_2_)(H_2_O)]_*n*_, the Pr^III^ ion is coordinated by five O atoms and one N atom from four benzimidazole-5,6-dicarboxyl­ate ligands, two O atoms from an acetate ligand and one water mol­ecule, giving a tricapped trigonal-prismatic geometry. The benzimidazole-5,6-dicarboxyl­ate and acetate ligands connect the Pr^III^ ions, forming a layer in the *ac* plane; the layers are further linked by N—H⋯O and O—H⋯O hydrogen bonding and π–π stacking inter­actions between neighboring pyridine rings [the centroid–centroid distance is 3.467 (1) Å], assembling a three-dimensional supra­molecular network. The acetate methyl group is disordered over two positions with site-occupancy factors of 0.75 and 0.25.

## Related literature

For related structures, see: Gao *et al.* (2008 ▶); Lo *et al.* (2007 ▶); Wang *et al.* (2009 ▶); Wei *et al.* (2008 ▶); Yao *et al.* (2008 ▶); Zhai (2009 ▶).
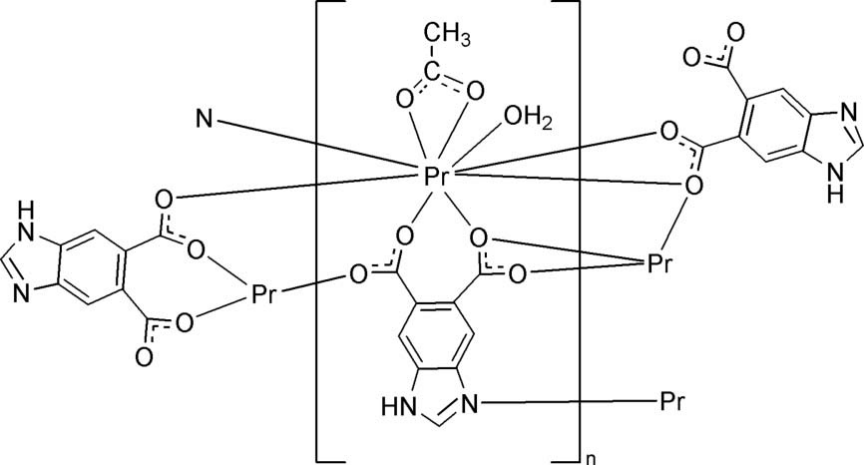


## Experimental

### 

#### Crystal data

[Pr(C_9_H_4_N_2_O_4_)(C_2_H_3_O_2_)(H_2_O)]
*M* *_r_* = 422.11
Triclinic, 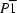

*a* = 7.4284 (5) Å
*b* = 9.0109 (7) Å
*c* = 9.7239 (7) Å
α = 87.075 (1)°
β = 86.498 (1)°
γ = 84.274 (1)°
*V* = 645.77 (8) Å^3^
*Z* = 2
Mo *K*α radiation
μ = 3.81 mm^−1^
*T* = 296 K
0.26 × 0.22 × 0.19 mm

#### Data collection

Bruker SMART APEX CCD diffractometer
Absorption correction: multi-scan (*SADABS*; Bruker, 2004 ▶) *T*_min_ = 0.386, *T*_max_ = 0.485
3963 measured reflections
2327 independent reflections
2184 reflections with *I* > 2σ(*I*)
*R*_int_ = 0.028

#### Refinement

*R*[*F* ^2^ > 2σ(*F* ^2^)] = 0.028
*wR*(*F* ^2^) = 0.072
*S* = 1.04
2327 reflections
199 parameters
22 restraints
H atoms treated by a mixture of independent and constrained refinement
Δρ_max_ = 1.23 e Å^−3^
Δρ_min_ = −1.45 e Å^−3^

### 

Data collection: *APEX2* (Bruker, 2004 ▶); cell refinement: *SAINT* (Bruker, 2004 ▶); data reduction: *SAINT*; program(s) used to solve structure: *SHELXS97* (Sheldrick, 2008 ▶); program(s) used to refine structure: *SHELXL97* (Sheldrick, 2008 ▶); molecular graphics: *SHELXTL* (Sheldrick, 2008 ▶); software used to prepare material for publication: *SHELXTL*.

## Supplementary Material

Crystal structure: contains datablocks I, global. DOI: 10.1107/S1600536810036986/pk2264sup1.cif

Structure factors: contains datablocks I. DOI: 10.1107/S1600536810036986/pk2264Isup2.hkl

Additional supplementary materials:  crystallographic information; 3D view; checkCIF report

## Figures and Tables

**Table 1:** Hydrogen-bond geometry (Å, °)

*D*—H⋯*A*	*D*—H	H⋯*A*	*D*⋯*A*	*D*—H⋯*A*
N1—H1⋯O6^i^	0.85 (2)	1.90 (3)	2.712 (5)	159 (5)
O1*W*—H1*W*⋯O2^ii^	0.83 (2)	2.06 (3)	2.854 (4)	159 (6)
O1*W*—H2*W*⋯O5^iii^	0.84 (2)	1.96 (2)	2.794 (4)	176 (5)

Symmetry codes: (i) 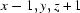; (ii) 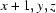; (iii) 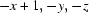.

**Fig. 1.**
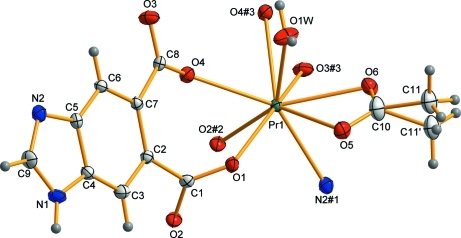
The asymmetric unit of the title compound, together with some symmetry related atoms to complete the coordination units. Displacement ellipsoids drawn at the 50% probability level. [Symmetry codes: (#1) x, y, -1+z; (#2) -x, 1-y, -z; (#3) 1-x, 1-y, -z.]

**Fig. 2.**
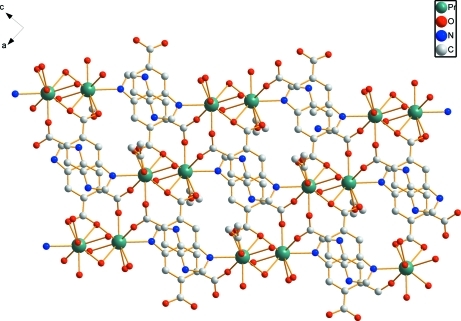
A view of the layers parallel to the ac plane.

## supplementary crystallographic information

### Comment

In recent years, studies of coordination polymers built using metals and multifunctional organic ligands, has been a rapidly expanding field. This has been due to their intriguing structural motifs and functional properties, such as molecular adsorption, magnetism, and luminescence. Benzimidazole-5,6-dicarboxylic acid (H_2_L) is such a multifuctional ligand with both nitrogen and oxygen donor atoms (Gao *et al.*, 2008; Lo *et al.*, 2007; Wang *et al.*, 2009; Wei *et al.*, 2008; Yao *et al.*, 2008; Zhai *et al.*, 2009). For example, Yao and co-workers have successfully synthesized six novel two-dimensional coordination polymers based on this ligand, namely, [MnL]_n_ (1), {[Ni_2_L_2_(H_2_O)_4_].(H_2_O)_3_}_n_ (2), {[Tb(L)(HL)(H_2_O)].(H_2_O)}_n_ (3) and {[Ln_2_L_2_(HL)_2_(H_2_O)_2_]}_n_ (Ln=Ho (4), Er (5), Lu (6)) (Yao *et al.*, 2008). Wei *et al.* also obtained a novel five coordinated Mn(II) polymer ([Mn(HL)])_n_ possessing abundant hydrogen bonds and π-π stacking interactions (Wei *et al.*, 2008). Herein, we report the hydrothermal synthesis, structure of the novel coordination polymer.

In the structure of the title compound (Fig. 1), each Pr^III^ centre is nine-coordinated by five oxygen atoms and one N atom from four benzimidazole-5,6-dicarboxylato ligands, two oxygen atoms from an acetate ligand, and one water molecule.  The structure can described as having a bicapped trigonal prismatic geometry with Pr···O distances and O···Pr···O angles ranging from 2.373 (3) Å to 2.645 (3) Å and 69.84 (9) ° to 152.88 (1) °, respectively.  The benzimidazole-5,6-dicarboxylate and acetate ligands, act as bridging ligands, linking the Pr^III^ metal centres into a layer parallel to the ac plane (Fig. 2).  Those layers are further connected via O—H···O and N—H···O hydrogen bonding interactions (Table 1) to form a three-dimensional supramolecular motif, which is stabilized by π-π stacking interactions between neighboring pyridyl rings (the centroid···centroid distance is 3.467 (1) Å).

### Experimental

A mixture of Pr_6_O_11_ (0.170 g; 0.17 mmol), benzimidazole-5,6-dicarboxylic acid (0.206 g; 1 mmol), acetic acid (0.06 g; 1 mmol), water (10 ml) was stirred vigorously for 30 min and then sealed in a teflon-lined stainless-steel autoclave (20 ml, capacity). The autoclave was heated and maintained at 423 K for 3 days, and then cooled to room temperature at 5 K h^-1^, which produced colorless block-shaped crystals.

### Refinement

Water H atoms were tentatively located in difference Fourier maps and were refined with distance restraints of O–H = 0.84 Å and H···H = 1.35 Å, and with *U*_iso_(H) = 1.5 *U*_eq_(O).  The H atom bound to the N1 nitrogen atom was refined with distance restraints of N–H = 0.86 Å, and with *U*_iso_(H) = 1.2 *U*_eq_(O).  All other H atoms were placed at calculated positions and treated as riding on the parent atoms, with C—H = 0.93 Å or 0.96 Å, and with *U*_iso_(H) = 1.2 or 1.5 *U*_eq_(C).

### Crystal data

**Table:**

[Pr(C_9_H_4_N_2_O_4_)(C_2_H_3_O_2_)(H_2_O)]	*Z* = 2
*M**_r_* = 422.11	*F*(000) = 408
Triclinic, *P*1	*D*_x_ = 2.171 Mg m^−^^3^
Hall symbol: -P 1	Mo *K*α radiation, λ = 0.71073 Å
*a* = 7.4284 (5) Å	Cell parameters from 2989 reflections
*b* = 9.0109 (7) Å	θ = 2.3–28.4°
*c* = 9.7239 (7) Å	µ = 3.81 mm^−^^1^
α = 87.075 (1)°	*T* = 296 K
β = 86.498 (1)°	Block, colourless
γ = 84.274 (1)°	0.26 × 0.22 × 0.19 mm
*V* = 645.77 (8) Å^3^	

### Data collection

**Table:**

Bruker SMART APEX CCD diffractometer	2327 independent reflections
Radiation source: fine-focus sealed tube	2184 reflections with *I* > 2σ(*I*)
graphite	*R*_int_ = 0.028
ω scans	θ_max_ = 25.2°, θ_min_ = 2.3°
Absorption correction: multi-scan (*SADABS*; Bruker, 2004)	*h* = −8→8
*T*_min_ = 0.386, *T*_max_ = 0.485	*k* = −10→10
3963 measured reflections	*l* = −7→11

### Refinement

**Table:**

Refinement on *F*^2^	Secondary atom site location: difference Fourier map
Least-squares matrix: full	Hydrogen site location: inferred from neighbouring sites
*R*[*F*^2^ > 2σ(*F*^2^)] = 0.028	H atoms treated by a mixture of independent and constrained refinement
*wR*(*F*^2^) = 0.072	*w* = 1/[σ^2^(*F*_o_^2^) + (0.0394*P*)^2^ + 1.0511*P*] where *P* = (*F*_o_^2^ + 2*F*_c_^2^)/3
*S* = 1.04	(Δ/σ)_max_ = 0.001
2327 reflections	Δρ_max_ = 1.23 e Å^−^^3^
199 parameters	Δρ_min_ = −1.45 e Å^−^^3^
22 restraints	Extinction correction: *SHELXL97* (Sheldrick, 2008), Fc^*^=kFc[1+0.001xFc^2^λ^3^/sin(2θ)]^-1/4^
Primary atom site location: structure-invariant direct methods	Extinction coefficient: 0.0091 (12)

### Special details

**Table:**

Geometry. All esds (except the esd in the dihedral angle between two l.s. planes) are estimated using the full covariance matrix. The cell esds are taken into account individually in the estimation of esds in distances, angles and torsion angles; correlations between esds in cell parameters are only used when they are defined by crystal symmetry. An approximate (isotropic) treatment of cell esds is used for estimating esds involving l.s. planes.
Refinement. Refinement of F^2^ against ALL reflections. The weighted R-factor wR and goodness of fit S are based on F^2^, conventional R-factors R are based on F, with F set to zero for negative F^2^. The threshold expression of F^2^ > 2sigma(F^2^) is used only for calculating R-factors(gt) etc. and is not relevant to the choice of reflections for refinement. R-factors based on F^2^ are statistically about twice as large as those based on F, and R- factors based on ALL data will be even larger.

### Fractional atomic coordinates and isotropic or equivalent isotropic displacement parameters (Å^2^)

**Table:**

	*x*	*y*	*z*	*U*_iso_*/*U*_eq_	Occ. (<1)
Pr1	0.35344 (3)	0.35784 (2)	−0.10574 (2)	0.01137 (13)	
O1	0.1245 (4)	0.2800 (3)	0.0551 (3)	0.0171 (6)	
O1W	0.6206 (5)	0.2207 (4)	0.0136 (4)	0.0286 (8)	
H1W	0.713 (5)	0.260 (5)	0.032 (6)	0.034*	
H2W	0.626 (7)	0.135 (3)	0.051 (6)	0.034*	
O2	−0.1348 (4)	0.4123 (3)	0.1093 (3)	0.0187 (7)	
O3	0.5275 (4)	0.4696 (4)	0.2957 (3)	0.0229 (7)	
O4	0.3620 (4)	0.4864 (3)	0.1165 (3)	0.0169 (6)	
O5	0.3704 (4)	0.0699 (3)	−0.1295 (3)	0.0213 (7)	
O6	0.5234 (4)	0.2005 (3)	−0.2842 (3)	0.0222 (7)	
N1	−0.1290 (5)	0.2444 (4)	0.6358 (4)	0.0197 (8)	
H1	−0.237 (4)	0.219 (6)	0.642 (6)	0.024*	
N2	0.1301 (5)	0.3045 (4)	0.7108 (4)	0.0183 (8)	
C1	0.0176 (5)	0.3514 (4)	0.1395 (4)	0.0124 (8)	
C2	0.0663 (5)	0.3516 (4)	0.2883 (4)	0.0128 (8)	
C3	−0.0669 (6)	0.3032 (5)	0.3815 (5)	0.0179 (9)	
H3	−0.1780	0.2820	0.3522	0.021*	
C4	−0.0292 (5)	0.2876 (5)	0.5200 (5)	0.0158 (9)	
C5	0.1336 (5)	0.3240 (4)	0.5684 (4)	0.0137 (8)	
C6	0.2660 (5)	0.3744 (4)	0.4746 (4)	0.0138 (8)	
H6	0.3747	0.3995	0.5056	0.017*	
C7	0.2352 (5)	0.3872 (4)	0.3345 (4)	0.0121 (8)	
C8	0.3828 (5)	0.4492 (4)	0.2436 (4)	0.0117 (8)	
C9	−0.0292 (6)	0.2583 (5)	0.7442 (5)	0.0191 (9)	
H9	−0.0696	0.2371	0.8348	0.023*	
C10	0.4743 (7)	0.0768 (5)	−0.2356 (6)	0.0304 (9)	
C11	0.5650 (11)	−0.0621 (7)	−0.2989 (8)	0.0304 (9)	0.75
H11A	0.6777	−0.0907	−0.2569	0.046*	0.75
H11B	0.5877	−0.0431	−0.3961	0.046*	0.75
H11C	0.4876	−0.1412	−0.2840	0.046*	0.75
C11'	0.477 (3)	−0.055 (2)	−0.332 (2)	0.0304 (9)	0.25
H11D	0.5368	−0.1430	−0.2891	0.046*	0.25
H11E	0.5413	−0.0309	−0.4178	0.046*	0.25
H11F	0.3551	−0.0717	−0.3491	0.046*	0.25

### Atomic displacement parameters (Å^2^)

**Table:**

	*U*^11^	*U*^22^	*U*^33^	*U*^12^	*U*^13^	*U*^23^
Pr1	0.01163 (17)	0.01259 (17)	0.00993 (17)	−0.00185 (9)	0.00044 (10)	−0.00098 (10)
O1	0.0192 (15)	0.0188 (15)	0.0137 (16)	−0.0041 (12)	0.0014 (12)	−0.0035 (12)
O1W	0.0286 (18)	0.0164 (16)	0.042 (2)	−0.0047 (14)	−0.0170 (16)	0.0049 (15)
O2	0.0167 (15)	0.0199 (15)	0.0190 (17)	0.0033 (12)	−0.0052 (13)	−0.0022 (13)
O3	0.0175 (16)	0.0315 (18)	0.0207 (18)	−0.0097 (13)	−0.0023 (13)	0.0039 (14)
O4	0.0200 (15)	0.0180 (15)	0.0131 (16)	−0.0055 (12)	0.0002 (12)	0.0008 (12)
O5	0.0240 (16)	0.0159 (15)	0.0239 (18)	−0.0040 (12)	0.0013 (14)	−0.0006 (13)
O6	0.0206 (16)	0.0198 (16)	0.0252 (18)	−0.0003 (12)	0.0048 (13)	−0.0019 (13)
N1	0.0150 (18)	0.029 (2)	0.016 (2)	−0.0078 (15)	0.0018 (15)	0.0025 (16)
N2	0.0203 (19)	0.0214 (18)	0.0130 (19)	−0.0036 (15)	0.0017 (15)	−0.0006 (15)
C1	0.0136 (19)	0.0106 (18)	0.013 (2)	−0.0035 (15)	0.0002 (16)	0.0017 (16)
C2	0.0128 (19)	0.0126 (19)	0.013 (2)	−0.0012 (15)	−0.0005 (16)	−0.0008 (16)
C3	0.014 (2)	0.022 (2)	0.018 (2)	−0.0019 (17)	−0.0023 (17)	−0.0015 (18)
C4	0.013 (2)	0.019 (2)	0.015 (2)	−0.0007 (16)	0.0010 (17)	−0.0014 (17)
C5	0.015 (2)	0.0135 (19)	0.012 (2)	−0.0001 (15)	0.0013 (16)	−0.0017 (16)
C6	0.0109 (19)	0.0138 (19)	0.017 (2)	−0.0015 (15)	0.0002 (16)	−0.0017 (17)
C7	0.0126 (19)	0.0108 (18)	0.013 (2)	−0.0006 (15)	0.0012 (16)	−0.0015 (16)
C8	0.015 (2)	0.0105 (18)	0.010 (2)	−0.0011 (15)	−0.0008 (16)	−0.0011 (15)
C9	0.022 (2)	0.025 (2)	0.010 (2)	−0.0049 (18)	0.0024 (17)	0.0032 (18)
C10	0.037 (2)	0.0221 (19)	0.031 (2)	0.0040 (18)	0.0026 (19)	−0.0054 (17)
C11	0.037 (2)	0.0221 (19)	0.031 (2)	0.0040 (18)	0.0026 (19)	−0.0054 (17)
C11'	0.037 (2)	0.0221 (19)	0.031 (2)	0.0040 (18)	0.0026 (19)	−0.0054 (17)

### Geometric parameters (Å, °)

**Table:**

Pr1—O1	2.373 (3)	N2—C9	1.310 (6)
Pr1—O6	2.498 (3)	N2—C5	1.386 (6)
Pr1—O2^i^	2.501 (3)	N2—Pr1^iv^	2.603 (4)
Pr1—O4	2.511 (3)	C1—C2	1.512 (6)
Pr1—O3^ii^	2.528 (3)	C2—C3	1.389 (6)
Pr1—O1W	2.541 (3)	C2—C7	1.429 (6)
Pr1—N2^iii^	2.603 (4)	C3—C4	1.389 (6)
Pr1—O5	2.606 (3)	C3—H3	0.9300
Pr1—O4^ii^	2.646 (3)	C4—C5	1.399 (6)
Pr1—C8^ii^	2.963 (4)	C5—C6	1.394 (6)
O1—C1	1.261 (5)	C6—C7	1.393 (6)
O1W—H1W	0.833 (19)	C6—H6	0.9300
O1W—H2W	0.836 (19)	C7—C8	1.499 (6)
O2—C1	1.253 (5)	C8—Pr1^ii^	2.963 (4)
O2—Pr1^i^	2.501 (3)	C9—H9	0.9300
O3—C8	1.250 (5)	C10—C11	1.503 (8)
O3—Pr1^ii^	2.528 (3)	C10—C11'	1.55 (2)
O4—C8	1.278 (5)	C11—H11A	0.9600
O4—Pr1^ii^	2.646 (3)	C11—H11B	0.9600
O5—C10	1.253 (6)	C11—H11C	0.9600
O6—C10	1.267 (6)	C11'—H11D	0.9600
N1—C9	1.342 (6)	C11'—H11E	0.9600
N1—C4	1.372 (6)	C11'—H11F	0.9600
N1—H1	0.85 (2)		
			
O1—Pr1—O6	125.76 (10)	C10—O6—Pr1	96.4 (3)
O1—Pr1—O2^i^	79.97 (10)	C9—N1—C4	107.0 (4)
O6—Pr1—O2^i^	135.37 (10)	C9—N1—H1	125 (4)
O1—Pr1—O4	69.81 (9)	C4—N1—H1	128 (4)
O6—Pr1—O4	147.25 (10)	C9—N2—C5	104.1 (4)
O2^i^—Pr1—O4	70.88 (10)	C9—N2—Pr1^iv^	122.6 (3)
O1—Pr1—O3^ii^	152.91 (11)	C5—N2—Pr1^iv^	133.1 (3)
O6—Pr1—O3^ii^	72.06 (11)	O2—C1—O1	123.2 (4)
O2^i^—Pr1—O3^ii^	73.82 (11)	O2—C1—C2	117.7 (4)
O4—Pr1—O3^ii^	106.94 (10)	O1—C1—C2	118.8 (3)
O1—Pr1—O1W	96.60 (11)	C3—C2—C7	120.7 (4)
O6—Pr1—O1W	74.37 (12)	C3—C2—C1	113.5 (4)
O2^i^—Pr1—O1W	145.00 (11)	C7—C2—C1	125.7 (4)
O4—Pr1—O1W	75.25 (11)	C2—C3—C4	118.0 (4)
O3^ii^—Pr1—O1W	108.74 (11)	C2—C3—H3	121.0
O1—Pr1—N2^iii^	84.24 (11)	C4—C3—H3	121.0
O6—Pr1—N2^iii^	71.46 (11)	N1—C4—C3	132.5 (4)
O2^i^—Pr1—N2^iii^	76.78 (11)	N1—C4—C5	104.9 (4)
O4—Pr1—N2^iii^	141.25 (11)	C3—C4—C5	122.5 (4)
O3^ii^—Pr1—N2^iii^	83.25 (11)	N2—C5—C6	130.8 (4)
O1W—Pr1—N2^iii^	137.92 (11)	N2—C5—C4	109.9 (4)
O1—Pr1—O5	75.84 (10)	C6—C5—C4	119.3 (4)
O6—Pr1—O5	50.87 (10)	C7—C6—C5	119.8 (4)
O2^i^—Pr1—O5	141.76 (10)	C7—C6—H6	120.1
O4—Pr1—O5	125.30 (10)	C5—C6—H6	120.1
O3^ii^—Pr1—O5	122.16 (11)	C6—C7—C2	119.7 (4)
O1W—Pr1—O5	67.81 (10)	C6—C7—C8	115.3 (3)
N2^iii^—Pr1—O5	71.75 (11)	C2—C7—C8	124.8 (4)
O1—Pr1—O4^ii^	139.88 (10)	O3—C8—O4	119.5 (4)
O6—Pr1—O4^ii^	86.63 (10)	O3—C8—C7	118.6 (4)
O2^i^—Pr1—O4^ii^	92.78 (10)	O4—C8—C7	121.9 (3)
O4—Pr1—O4^ii^	70.53 (11)	O3—C8—Pr1^ii^	57.8 (2)
O3^ii^—Pr1—O4^ii^	49.85 (9)	O4—C8—Pr1^ii^	63.2 (2)
O1W—Pr1—O4^ii^	67.41 (10)	C7—C8—Pr1^ii^	165.0 (3)
N2^iii^—Pr1—O4^ii^	132.75 (10)	N2—C9—N1	114.0 (4)
O5—Pr1—O4^ii^	124.59 (9)	N2—C9—H9	123.0
O1—Pr1—C8^ii^	159.06 (10)	N1—C9—H9	123.0
O6—Pr1—C8^ii^	75.06 (10)	O5—C10—O6	121.2 (4)
O2^i^—Pr1—C8^ii^	85.58 (10)	O5—C10—C11	121.3 (5)
O4—Pr1—C8^ii^	91.20 (10)	O6—C10—C11	117.1 (5)
O3^ii^—Pr1—C8^ii^	24.71 (10)	O5—C10—C11'	115.5 (10)
O1W—Pr1—C8^ii^	86.57 (11)	O6—C10—C11'	119.2 (10)
N2^iii^—Pr1—C8^ii^	107.21 (11)	C10—C11—H11A	109.5
O5—Pr1—C8^ii^	124.02 (10)	C10—C11—H11B	109.5
O4^ii^—Pr1—C8^ii^	25.55 (10)	H11A—C11—H11B	109.5
C1—O1—Pr1	131.7 (3)	C10—C11—H11C	109.5
Pr1—O1W—H1W	124 (3)	H11A—C11—H11C	109.5
Pr1—O1W—H2W	127 (3)	H11B—C11—H11C	109.5
H1W—O1W—H2W	108 (3)	C10—C11'—H11D	109.5
C1—O2—Pr1^i^	148.2 (3)	C10—C11'—H11E	109.5
C8—O3—Pr1^ii^	97.5 (2)	H11D—C11'—H11E	109.5
C8—O4—Pr1	137.6 (3)	C10—C11'—H11F	109.5
C8—O4—Pr1^ii^	91.2 (2)	H11D—C11'—H11F	109.5
Pr1—O4—Pr1^ii^	109.47 (11)	H11E—C11'—H11F	109.5
C10—O5—Pr1	91.6 (3)		
			
O6—Pr1—O1—C1	−171.8 (3)	O2—C1—C2—C3	49.4 (5)
O2^i^—Pr1—O1—C1	−32.1 (3)	O1—C1—C2—C3	−124.8 (4)
O4—Pr1—O1—C1	41.1 (3)	O2—C1—C2—C7	−134.6 (4)
O3^ii^—Pr1—O1—C1	−46.8 (5)	O1—C1—C2—C7	51.3 (6)
O1W—Pr1—O1—C1	112.7 (4)	C7—C2—C3—C4	−1.4 (6)
N2^iii^—Pr1—O1—C1	−109.6 (4)	C1—C2—C3—C4	174.8 (4)
O5—Pr1—O1—C1	177.8 (4)	C9—N1—C4—C3	−176.2 (5)
O4^ii^—Pr1—O1—C1	50.3 (4)	C9—N1—C4—C5	1.1 (5)
C8^ii^—Pr1—O1—C1	15.0 (5)	C2—C3—C4—N1	179.5 (4)
O1—Pr1—O4—C8	58.8 (4)	C2—C3—C4—C5	2.5 (6)
O6—Pr1—O4—C8	−66.6 (4)	C9—N2—C5—C6	178.5 (4)
O2^i^—Pr1—O4—C8	144.9 (4)	Pr1^iv^—N2—C5—C6	3.1 (7)
O3^ii^—Pr1—O4—C8	−149.6 (4)	C9—N2—C5—C4	0.0 (5)
O1W—Pr1—O4—C8	−44.1 (4)	Pr1^iv^—N2—C5—C4	−175.3 (3)
N2^iii^—Pr1—O4—C8	109.9 (4)	N1—C4—C5—N2	−0.7 (5)
O5—Pr1—O4—C8	4.3 (4)	C3—C4—C5—N2	177.0 (4)
O4^ii^—Pr1—O4—C8	−114.9 (4)	N1—C4—C5—C6	−179.3 (4)
C8^ii^—Pr1—O4—C8	−130.2 (3)	C3—C4—C5—C6	−1.7 (6)
O1—Pr1—O4—Pr1^ii^	173.75 (13)	N2—C5—C6—C7	−178.6 (4)
O6—Pr1—O4—Pr1^ii^	48.4 (2)	C4—C5—C6—C7	−0.3 (6)
O2^i^—Pr1—O4—Pr1^ii^	−100.20 (12)	C5—C6—C7—C2	1.3 (6)
O3^ii^—Pr1—O4—Pr1^ii^	−34.66 (13)	C5—C6—C7—C8	177.3 (4)
O1W—Pr1—O4—Pr1^ii^	70.84 (12)	C3—C2—C7—C6	−0.4 (6)
N2^iii^—Pr1—O4—Pr1^ii^	−135.22 (14)	C1—C2—C7—C6	−176.2 (4)
O5—Pr1—O4—Pr1^ii^	119.17 (11)	C3—C2—C7—C8	−176.0 (4)
O4^ii^—Pr1—O4—Pr1^ii^	0.0	C1—C2—C7—C8	8.2 (6)
C8^ii^—Pr1—O4—Pr1^ii^	−15.29 (12)	Pr1^ii^—O3—C8—O4	14.5 (4)
O1—Pr1—O5—C10	169.4 (3)	Pr1^ii^—O3—C8—C7	−163.1 (3)
O6—Pr1—O5—C10	0.2 (3)	Pr1—O4—C8—O3	107.5 (4)
O2^i^—Pr1—O5—C10	117.0 (3)	Pr1^ii^—O4—C8—O3	−13.7 (4)
O4—Pr1—O5—C10	−138.6 (3)	Pr1—O4—C8—C7	−75.0 (5)
O3^ii^—Pr1—O5—C10	11.5 (3)	Pr1^ii^—O4—C8—C7	163.8 (3)
O1W—Pr1—O5—C10	−87.3 (3)	Pr1—O4—C8—Pr1^ii^	121.2 (3)
N2^iii^—Pr1—O5—C10	80.8 (3)	C6—C7—C8—O3	7.4 (5)
O4^ii^—Pr1—O5—C10	−49.0 (3)	C2—C7—C8—O3	−176.9 (4)
C8^ii^—Pr1—O5—C10	−18.0 (3)	C6—C7—C8—O4	−170.1 (4)
O1—Pr1—O6—C10	−13.2 (3)	C2—C7—C8—O4	5.6 (6)
O2^i^—Pr1—O6—C10	−128.3 (3)	C6—C7—C8—Pr1^ii^	−64.3 (11)
O4—Pr1—O6—C10	96.2 (3)	C2—C7—C8—Pr1^ii^	111.4 (10)
O3^ii^—Pr1—O6—C10	−170.2 (3)	C5—N2—C9—N1	0.7 (5)
O1W—Pr1—O6—C10	73.6 (3)	Pr1^iv^—N2—C9—N1	176.7 (3)
N2^iii^—Pr1—O6—C10	−81.4 (3)	C4—N1—C9—N2	−1.2 (5)
O5—Pr1—O6—C10	−0.2 (3)	Pr1—O5—C10—O6	−0.4 (5)
O4^ii^—Pr1—O6—C10	141.1 (3)	Pr1—O5—C10—C11	171.3 (5)
C8^ii^—Pr1—O6—C10	164.2 (3)	Pr1—O5—C10—C11'	−157.3 (10)
Pr1^i^—O2—C1—O1	−128.3 (5)	Pr1—O6—C10—O5	0.4 (5)
Pr1^i^—O2—C1—C2	57.8 (7)	Pr1—O6—C10—C11	−171.7 (5)
Pr1—O1—C1—O2	88.8 (5)	Pr1—O6—C10—C11'	156.5 (11)
Pr1—O1—C1—C2	−97.4 (4)		

Symmetry codes: (i) −*x*, −*y*+1, −*z*; (ii) −*x*+1, −*y*+1, −*z*; (iii) *x*, *y*, *z*−1; (iv) *x*, *y*, *z*+1.

### Hydrogen-bond geometry (Å, °)

**Table:**

*D*—H···*A*	*D*—H	H···*A*	*D*···*A*	*D*—H···*A*
N1—H1···O6^v^	0.85 (2)	1.90 (3)	2.712 (5)	159 (5)
O1W—H1W···O2^vi^	0.83 (2)	2.06 (3)	2.854 (4)	159 (6)
O1W—H2W···O5^vii^	0.84 (2)	1.96 (2)	2.794 (4)	176 (5)

Symmetry codes: (v) *x*−1, *y*, *z*+1; (vi) *x*+1, *y*, *z*; (vii) −*x*+1, −*y*, −*z*.
